# Multicenter Clinical Evaluation of BacT/Alert Virtuo Blood Culture System

**DOI:** 10.1128/JCM.00307-17

**Published:** 2017-07-25

**Authors:** Michael R. Jacobs, Tony Mazzulli, Kevin C. Hazen, Caryn E. Good, Ayman M. Abdelhamed, Pauline Lo, Bianche Shum, Katharine P. Roman, Danielle C. Robinson

**Affiliations:** a Department of Pathology, Case Western Reserve University and University Hospitals Cleveland Medical Center, Cleveland, Ohio, USA; b Department of Laboratory Medicine and Pathobiology, University of Toronto, Toronto, ON, Canada; c Department of Microbiology, Mount Sinai Hospital/University Health Network, Toronto, ON, Canada; d Department of Pathology, Clinical Microbiology Laboratory, Duke University, Durham, North Carolina, USA

**Keywords:** Virtuo, blood culture

## Abstract

BacT/Alert Virtuo is an advanced, automated blood culture system incorporating improved automation and an enhanced detection algorithm to shorten time to detection. A multicenter study of the investigational Virtuo system (bioMérieux, Inc., Durham, NC) compared to BacT/Alert 3D (BTA3D) for detection of bacteremia/fungemia in four bottle types, SA and FA Plus (aerobic) and SN and FN Plus (anaerobic), was performed in a clinical setting with patient samples in a matched system design clinical trial. Blood was added to paired aerobic or anaerobic bottles, with the volume in each bottle in each pair required to be ≤10 ml and with the volumes required to be within 30% of each other. Of 5,709 bottle sets (52.5% aerobic pairs and 47.5% anaerobic pairs), 430 (7.5%) were positive for bacterial or fungal growth, with 342 (6.0%) clinically significant and 83 (1.5%) contaminated. A total of 3,539 sets (62.0%) were volume compliant, with 203 sets (5.7%) clinically significant. The positivity rates for volume-compliant bottle pairs determined by the two systems were comparable, with 68.7% of clinically significant isolates detected by both instruments, 15.7% by Virtuo only, and 15.7% by BTA3D only. Virtuo detected microbial growth nearly 2 h sooner overall than BTA3D (mean, 15.9 h versus 17.7 h). Shorter time to detection by Virtuo was related to organism group, with the time to detection being significantly shorter for enteric Gram-negative bacilli and enterococci (means, 3.6 h and 2.3 h shorter, respectively). This large clinical study demonstrated that the Virtuo blood culture system produced results comparable to those seen with the long-established BTA3D system, with significantly shorter time to detection.

## INTRODUCTION

Detection of microorganisms in blood in clinical microbiology laboratories is important for the prompt isolation, identification, and determination of antimicrobial susceptibility of bacterial and fungal isolates, allowing clinicians to optimize treatment (1). Staphylococcus aureus, Escherichia coli, Enterococcus spp., Klebsiella pneumoniae, coagulase-negative staphylococci, Pseudomonas aeruginosa, Candida albicans, Enterobacter cloacae, and Serratia marcescens continue to be the pathogens most frequently recovered from patients with sepsis (2). Intravenous catheters are the most common primary source of bloodstream infection, responsible for around one-quarter of episodes of sepsis, with over 80% of bloodstream infections acquired in health care institutions (2). Crude and attributable mortality rates for sepsis are high, at 20% and 12%, respectively, with higher mortality associated with increasing age, hypotension, absence of fever, hospital acquisition, high white blood cell count, presence of AIDS, malignancy, or renal disease (2).

As the microorganism load of most cases of bacteremia and fungemia is frequently low and intermittent, blood culture procedures should be designed to overcome these factors, as well as to overcome the inhibitory effect of antimicrobial agents and components of blood. Variables affecting blood culture yields include the volume of blood cultured, the number of cultures performed, the culture media used, the detection methods, and the length of incubation (3). Media containing polymeric beads that adsorb antimicrobials, such as BacT/Alert FA Plus (aerobic) and FN Plus (anaerobic) media, have been shown to provide improved recovery and early detection of microorganisms compared with charcoal-containing BacT/Alert FA and FN media (4), with shorter detection times (5).

The importance of blood cultures is emphasized in recent evidence-based recommendations for improving the outcome of sepsis, referred to as the “Surviving Sepsis Campaign” (6, 7). Evidence-based recommendations made regarding many aspects of the management of acute sepsis and septic shock include performing blood cultures as clinically appropriate before initiating antimicrobial therapy and collection of at least two sets of blood cultures (using both aerobic and anaerobic bottles), with at least one drawn through each vascular access device, unless the device had been inserted recently (<48 h), and one drawn percutaneously from a peripheral vein. The recommended volume of each blood draw should be ≥10 ml (6).

The BacT/Alert system (bioMérieux, Inc., Durham, NC) is an automated microbial detection system introduced into clinical use in 1990 based on colorimetric detection by pH sensors of CO_2_ produced by growing microorganisms (8). The bottles and instruments used in the BacT/Alert system have evolved since their initial introduction, with development of new media and instruments, with various generations of the BacT/Alert 3D (BTA3D) instrument in use since 1998. A more advanced and automated instrument, BacT/Alert Virtuo, was introduced in 2014 in Europe (9). Virtuo uses the same detection principle as the BTA3D using BacT/Alert reagent bottles. Enhancements of Virtuo over previous BTA3D generations include incorporation of new instrument architecture to improve temperature stability, workflow improvement via automation of processes that are currently performed manually, an improved user interface, and an enhanced proprietary algorithm to shorten time to detection of positive cultures. A single Virtuo instrument holds 428 bottles, and up to four instruments can be attached together with a common loading area. The loading bay is motion activated with optical sensors to scan and automatically load BacT/Alert bottles via a conveyor belt and internal robotic arm. The scanning station rotates and images the entire BacT/Alert bottle for definitive identification from bar-coded labels. Positive bottles are unloaded into an external chute automatically or on demand and negative bottles into a removable waste container at the end of the culture period. The instrument's software has automated detector cell quality control and calibration and may be connected to a laboratory information system or to Myla Microbiology middleware (bioMérieux).

This multicenter study evaluated the performance of the investigational Virtuo system for detection of bacteremia and fungemia in four bottle types using prospectively collected patient blood samples tested in parallel with the BTA3D system. The primary objective of the study was to show that the Virtuo blood culture system produced results comparable to those produced by the long-established BTA3D system using the same blood culture bottle types.

## RESULTS

Three institutions, University Hospitals Cleveland Medical Center (Cleveland, OH), Mt. Sinai Hospital (Toronto, Ontario, Canada), and Duke University Medical Center (Durham, NC), participated in the study, testing a total of 5,709 bottle pairs. All positive and negative daily controls produced appropriate results. Descriptive statistics on the bottle types included in the study, broken down by location and bottle type, are shown in Table 1. Overall, 52.5% of the bottles in the bottle pairs were aerobic bottles (22.7% were SA and 29.8% were FA Plus bottles) and 47.5% were anaerobic bottles (22.0% were SN and 25.5% were FN Plus bottles). Overall, 3,539 (62.0%) of the 5,709 bottle pairs were within the blood volume compliance limits defined by the protocol; i.e., those bottles contained ≤10 ml each, with the volume in the bottle with the lower volume in each pair within 30% of that in the bottle with the higher volume. Among the volume-noncompliant bottle sets, 1,165 (20.4%) contained ≤10 ml of blood in each bottle but with volumes that were outside the 30% volume agreement between the two bottles, and another 1,005 (17.6%) had blood volumes of greater than 10 ml in one or both bottles (Table 2). The mean volumes per bottle were 4.8 ml (Virtuo) and 4.8 ml (BTA3D) in the volume-compliant sets and 12.1 ml and 12.2 ml, respectively, where fill volume exceeded 10 ml per bottle in one or both bottles of each pair.

**TABLE 1:** Number of bottle pairs tested by site by bottle type and compliance with blood volume requirements

Study site	No. of bottle sets by bottle type (% vol-compliant sets)				
	SA	SN	FA Plus	FN Plus	Total
A	1,297 (58.1)	1,256 (65.8)	384 (64.1)	597 (65.2)	3,534 (62.7)
B	—^*a*^	—	426 (69.5)	166 (80.1)	592 (72.5)
C	—	—	892 (57.3)	691 (55.6)	1,583 (56.5)
Total	1,297 (58.1)	1,256 (65.8)	1,702 (61.9)	1,454 (62.3)	5,709 (62.0)

a —, bottle type not used by site.

**TABLE 2:** Percentages of bottle pairs tested that were volume compliant and noncompliant by bottle type

Bottle type (*n*)	Volume-compliant/non-volume-compliant bottle pairs (%)		
	Compliant	Noncompliant (vol = ≤10 ml)	Noncompliant (vol = >10 ml)
SA (1,297)	58.1	20.6	21.3
SN (1,256)	65.8	18.7	15.5
FA Plus (1,702)	61.9	22.8	15.3
FN Plus (1,454)	62.3	18.9	18.8
All (5,709)	62.0	20.4	17.6

A total of 430 (7.5%) of the 5,709 bottle sets were positive for bacterial or fungal growth, with 342 classified as clinically significant, 83 (1.5%) contaminated, and 5 (0.1%) indeterminate (Table 3). A total of 203 sets were both clinically significant and volume compliant. Several sets had multiple significant organisms, with the 203 volume-compliant sets yielding 217 significant bacterial isolates (Tables 4 and 5). An additional 139 clinically significant positive sets were detected in volume-noncompliant bottle pairs.

**TABLE 3:** Positivity rates of all bottle types combined according to volume compliance status and clinical significance of bottles with positive results^*a*^

Volume compliance status	Total no. of bottle pairs	No. (%) of bottles			
		All positives	Clinically significant positives	Contaminated	Indeterminate
Compliant	3,539	257 (7.3)	203 (5.7)	49 (1.4)	5 (0.1)
Noncompliant, ≤10 ml	1,165	86 (7.4)	64 (5.5)	22 (1.9)	0
Noncompliant, >10 ml	1,005	87 (8.7)	75 (7.5)^*b*^	12 (1.2)	0
All	5,709	430 (7.5)	342 (6.0)	83 (1.5)	5 (0.1)

a Positive culture data imply that at least one bottle from the bottle pair gave a positive signal and had a positive subculture.

b Data were statistically significant compared to rate determined for volume-compliant bottle sets by Pearson's chi-square test (*P* = 0.044).

**TABLE 4:** Percent positivity rates, analyzed by bottle type and volume compliance status^*a*^

Bottle type	Compliant				Noncompliant, ≤10-ml vol				Noncompliant, >10-ml vol			
	No. of bottle pairs	% positives	% significant positives	% contaminated	No. of bottle pairs	% positives	% significant positives	% contaminated	No. of bottle pairs	% positives	% significant positives	% contaminated
SA	754	7.3	5.6	1.6	267	7.5	4.5	3.0	276	5.8	4.7	1.1
SN	826	5.0	4.2	0.6	235	4.3	3.0	1.3	195	3.1	3.1	0.0
FA Plus	1,053	8.9	7.0	1.6	388	7.7	5.9	1.8	261	14.2^*b*^	11.9^*b*^	2.3
FN Plus	906	7.4	5.7	1.7	275	9.5	8.0	1.5	273	10.3	9.2^*c*^	1.1
All	3,539	7.3	5.7	1.4	1,165	7.4	5.5	1.9	1,005	8.7	7.5	1.2

a Positive culture results imply that at least one bottle from a bottle pair gave a positive result and a positive subculture was obtained. Data for 5 indeterminate positives, all in volume-compliant bottle sets, 1 each in SA and SN and 3 in FA Plus, were excluded from this table.

b Data were statistically significant by Pearson's chi-square test; *P* = 0.011 (all positives) and *P* = 0.010 (significant positives).

c Data were statistically significant; *P* = 0.045.

**TABLE 5:** Numbers of clinically significant isolates detected, analyzed by bottle type and instrument detection for volume-compliant and noncompliant groups^*a*^

Bottle type	No. of bottles with indicated volume compliance and assay status											
	Compliant				Noncompliant, ≤10-ml vol				Noncompliant, >10-ml vol			
	Significant positives	Both Virtuo and BTA3D	Virtuo only	BTA3D only	Significant positives	Both Virtuo and BTA3D	Virtuo only	BTA3D only	Significant positives	Both Virtuo and BTA3D	Virtuo only	BTA3D only
SA	45	34	6	5	12	7	1	4	13	7	4	2
SN	39	23	9	7	7	5	2	0	7	1	5	1
FA Plus	78	52	12	14	23	12	7	4	32	21	6	5
FN Plus	55	40	7	8	23	15	4	4	27	19	4	4
All	217	149	34	34	65	39	14	12	79	48	19	12

a A total of 217 clinically significant positive isolates were recovered from 203 clinically significant bottle sets. No statistical difference was found in rates of detection between volume-compliant bottles and either type of non-volume-compliant bottle (*P* = 0.194 and *P* = 0.202, respectively).

An overall positivity rate of 7.3% was found among the volume-compliant bottle sets, with 5.7% clinically significant and 1.4% with contaminants (Table 4). Positivity rates for all noncompliant bottle sets with blood volumes of ≤10 ml and for SA and SN bottles with blood volumes of >10 ml were similar to those of the compliant sets. The positivity rates of clinically significant pathogens in noncompliant FA Plus and FN Plus bottle sets with blood volumes of >10 ml were significantly higher than those seen with the compliant bottle sets (*P* = 0.010 for FA Plus and *P* = 0.045 for FN Plus).

Numbers of clinically significant bacterial and fungal isolates are reported according to their detection in either or both of the two test instruments in Table 5. Of the 217 clinically significant isolates detected in volume-compliant bottle sets, 149 (68.7%) were detected by both instruments, while 34 were detected only by Virtuo and 34 were detected only by BTA3D. The noncompliant sets showed similar rates of positivity, with 39 (60.0%) of 65 clinically significant isolates detected by both instruments in the ≤10-ml group and 48 (60.8%) of 79 in the >10-ml group. The differences between these volume-compliant and noncompliant positivity rates were not statistically significant (*P* = 0.194 versus the ≤10-ml volume group and *P* = 0.202 versus the >10-ml volume group), nor was there a difference noted between these groups in the single-machine detection rates.

The microorganism groups detected in the volume-compliant group and both noncompliant groups are shown in Table 6, with a more detailed organism listing shown in Table S1 in the supplemental material. Clinically significant isolates recovered from volume-compliant bottles included 66 Enterobacteriaceae isolates, 45 S. aureus isolates, 31 coagulase-negative staphylococcal isolates, 13 streptococcal isolates, 29 enterococcal isolates, 14 nonfermentative Gram-negative bacillus isolates, 8 yeast isolates (all Candida species), 7 anaerobe isolates, and 4 other isolates. Overall distributions of pathogens were similar in volume-noncompliant bottles, and the overall rate of detection by both systems was not significantly lower (60.0 to 60.8%) than that seen with the compliant group (see Table 5). However, the rate of isolation of Candida species was higher in bottles inoculated with >10 ml (19/1,005; 1.9%) than in those inoculated with ≤10 ml (10/4,704; 0.2%) (*P* < 0.0001) (Table 6). No differences were noted between instruments for bottle pairs where only one of the pair had organisms detected (Table 6; see also Table S1 in the supplemental material).

**TABLE 6:** Numbers of clinically significant isolates by organism type and recognition by both instruments, Virtuo and BTA3D, for volume-compliant and noncompliant groups^*a*^

Organism group	No. of isolates with indicated volume compliance status					
	Compliant		Noncompliant, ≤10-ml vol		Noncompliant, >10-ml vol	
	Significant positives	Detected by both instruments^*b*^	Significant positives	Detected by both instruments^*c*^	Significant positives	Detected by both instruments^*d*^
Coagulase-negative staphylococci	31	17	12	7	9	5
Staphylococcus aureus	45	31	15	11	12	9
Enterobacteriaceae	66	50	21	10	14	10
Yeast	8	6	2	1	19^*e*^	10
Nonfermentative Gram-negative bacilli	14	8	0	0	5	2
Anaerobes	7	1	0	0	1	1
Streptococcus spp.	13	10	11	7	9	7
Enterococcus spp.	29	24	4	3	9	4
Other	4	2	0	0	1	0
Total for all microorganisms	217	149	65	39	79	48

a A detailed organism listing is shown in Table S1 in the supplemental material.

b Rate of detection by both instruments, 68.7% (detection rate differences not statistically significant; see Table 5).

c Rate of detection by both instruments, 60.0% (detection rate differences not statistically significant; see Table 5).

d Rate of detection by both instruments, 60.8% (detection rate differences not statistically significant; see Table 5).

e Significant versus combined volume-compliant and non-volume-noncompliant ≤10-ml-volume groups (*P* < 0.0001).

There were significant differences between the two instruments in mean and median times to microbial detection (TTD) for volume-compliant bottle pairs (Table 7). Overall, the Virtuo system detected microbial growth nearly 2 h sooner overall than the BTA3D system (15.9 h versus 17.7 h). Differences in TTD were related to organism group, being significant for enteric Gram-negative bacilli and enterococci (Virtuo mean TTD, 3.6 h and 2.3 h sooner, respectively) but not for other organism groups. Differences were not associated with fill volumes, which were comparable (mean, 4.4 ml in bottles tested in the Virtuo system versus 4.3 ml in BTA3D).

**TABLE 7:** Mean and median time to microbial detection for volume-compliant bottle sets by Virtuo and BTA3D and mean bottle fill volumes for clinically significant isolates recovered from both study bottles in each pair^*a*^

Organism group	*n*	Mean TTD (h)		*P* value^*b*^	Median TTD (h)		*P* value^*c*^	Mean fill, ml	
		Virtuo	3D		Virtuo	BTA3D		Virtuo	BTA3D
Coagulase-negative staphylococci	14	20.6	21.4	0.678	19.4	20.8	0.078	4.1	3.9
Enteric Gram-negative bacilli	43	11.1	14.7	<0.001	8.6	11.0	<0.001	4.7	4.6
Enterococcus spp.	18	10.6	12.9	<0.001	11.4	13.4	<0.001	4.3	4.4
Staphylococcus aureus	30	20.8	20.5	0.819	16.1	19.2	0.580	4.7	4.6
Streptococcus spp.	9	11.7	14.5	0.221	8.6	10.4	0.098	2.3	2.6
Yeast	4	26.7	26.4	0.936	26.5	27.7	0.875	4.8	5.0
Other	1	93.6	96.0	NA	93.6	96.0	1.000	0.8	1.0
All	119	15.9	17.7	0.001	12.2	13.4	<0.001	4.4	4.3

a Results shown are for bottle sets where clinically significant, monomicrobial isolates were recovered from both bottles. Six outliers with a difference in time to microbial detection (TTD) of ≥24 h, suggestive of greatly different organism loads at the time of bottle inoculation, were excluded from this analysis.

b Data represent TTD means. A *t* test was used to evaluate the mean of the differences.

c Data represent TTD medians. A signed-rank test was used to evaluate the median of the differences in TTD since the TTD data were not normally distributed.

Subculture of bottles signaling positively on instruments and containing up to 10 ml of blood (as specified in the fill recommendations in the instructions for the use of bottles) showed 4 false positives from Virtuo (4/3,235, 0.12%) and 11 from BTA3D (11/2,982, 0.37%). Subculture of negative bottles from bottle sets where one bottle was positive showed two false-negative results in Virtuo bottles (Propionibacterium acnes and C. albicans) and three in BTA3D bottles (S. aureus, coagulase-negative staphylococcus, and Acinetobacter radioresistens). Subculture of approximately two-thirds of the negative bottle pairs showed 12/3,169 (0.38%) false negatives in the Virtuo system and 8/2,917 (0.27%) in the BTA3D system (*P* = 0.477). Virtuo false negatives comprised four P. acnes isolates, five Candida species isolates (one C. albicans, two C. glabrata, and two C. parapsilosis), and one each of K. pneumoniae, C. perfringens, and coagulase-negative staphylococcus isolates. BTA3D false negatives included four P. acnes and four Candida species (one C. albicans, one C. glabrata, and two C. parapsilosis). In all cases where isolates were considered pathogens, the same organism was recovered from other blood cultures collected within 24 h of the false-negative occurrence.

## DISCUSSION

In our large-scale study of 5,709 bottle pairs, the Virtuo system met the primary endpoint of equivalence to the BTA3D predicate device, with comparable levels of pathogen recovery by the two systems. Advantages of the Virtuo system include improved automation, including mechanical loading and unloading of bottles, potentially leading to labor savings, and a significantly shorter time to detection of enteric Gram-negative bacilli (mean, 11.1 h versus 14.7 h; median, 8.6 h versus 11.4 h) and enterococci (mean, 10.6 h versus 12.9 h; median, 11.4 h versus 13.4 h). As enteric Gram-negative bacilli include carbapenem-resistant species, this finding is particularly notable for situations in which rapid diagnostic methods are performed directly from positive blood cultures, further shortening the overall turnaround time to identification and to determinations of antimicrobial susceptibility.

The yield of positive blood cultures is directly related to the blood volume cultured, with larger-volume draws consistently shown to be superior to lower-volume draws (10, 11). As the blood culture bottles used in the BTA3D system are approved for blood draws of up to 10 ml, three bottles per draw are recommended for optimal yield (12). In our study, the blood volume in at least one bottle of each bottle pair exceeded 10 ml in 1,005 of the 5,709 bottle pairs. Pathogen recovery was significantly higher in these high-volume sets (7.5%) than in the volume-compliant group (5.7% and 5.5%; *P* = 0.044) (Table 3), with this difference associated with use of FA Plus and FN Plus bottles (Table 4). Recovery of Candida species was also higher from high-volume sets. We also noted that mean blood volumes per bottle in compliant bottle pairs containing the same spectrum of clinically significant isolates were suboptimal, with means of 4.4 ml and 4.3 ml in bottles incubated in the Virtuo and BTA3D systems, respectively (Table 7).

The value of adsorbent polymeric beads for neutralization of antimicrobials in the FA Plus and FN Plus bottles in improving organism yield was also demonstrated in our study. Rates of identification of significant positive isolates with Plus bottles were consistently higher in the volume-compliant group as well as in both volume-noncompliant groups (Table 4). Differences were greatest for the highest-volume group (>10 ml), with a significant recovery rate of 11.9% in FA Plus bottles compared to 4.7% in SA bottles and 9.2% in FN Plus bottles versus 3.1% in SN bottles.

Detection of pathogens in both of the bottles in a pair versus in one bottle showed no association between Virtuo and BTA3D instruments and no association between volume compliance groups (Table 6; see also Table S1 in the supplemental material). These findings suggest that the predominant factors associated with recovery of an organism from one bottle of a pair are primarily those of low organism load in blood specimens and the presence of antimicrobial agents.

Our findings are in agreement with those from a limited study of simulated blood cultures comparing the Virtuo blood culture system to the BTA3D system (9). The detection rates for 115 clinical bacterial and fungal isolates in 784 simulated blood culture bottles did not differ, while the median time to detection was significantly reduced from 15 h in the BTA3D system to 12 h in the Virtuo system (*P* < 0.0001), without striking differences noted between bacterial species or bottle types. In this study, 90% of bottles inoculated with bacteria reached positivity within 16 h in the Virtuo system compared with 21 h in the BTA3D. Differences in time to detection between the systems were not seen for all organism groups in our study, and the explanation is likely associated with factors not present in the simulated study such as lower bacterial loads and presence of antimicrobial agents in patient specimens.

In our study, operators using the Virtuo system appreciated the benefits of the decreased handling time for bottles resulting from bottle identification by the use of automation for scanning of bottle labels and loading and unloading of bottles. Although not tested in our study, the Virtuo system also has the ability to calculate bottle fill volumes by scanning the bottle and determining the fill volume from the scanned liquid level, which will allow users to comply with the standard of the College of American Pathologists to monitor blood culture fill volumes from adult patients and to provide feedback to clinical staff (CAP Standard MIC.22640).

Our study had several limitations. These included a high proportion of volume-noncompliant bottles or bottle sets and the fact that predominantly only one additional bottle (aerobic or anaerobic) was used rather than two additional bottles (both aerobic and anaerobic) in each set. However, our findings showed that results from volume-noncompliant sets were similar to those from compliant sets, with a higher organism yield from high-volume bottles.

While many rapid, non-culture-based diagnostic methods based on PCR, mass spectroscopy, and microarrays are in development for identification of pathogens and major antimicrobial resistance determinants, clinical experience with these methods is limited, and standard blood culture remains the standard of care (7). One non-culture-based system, a direct-from-blood PCR-based system, the T2 Candida Panel, is approved for clinical use and enables detection of Candida directly from blood, but its use is limited by the low yield of this target in sepsis (13). Another approach is for rapid identification of positive blood culture bottles using PCR, peptide nucleic acid-fluorescent in situ hybridization (PNA-FISH), matrix-assisted laser desorption ionization–time of flight (MALDI-TOF) mass spectroscopy, and other techniques. The shorter detection time of the Virtuo system will allow earlier implementation of these techniques.

In conclusion, this study met its primary objective of showing that the Virtuo blood culture system produced results comparable to those produced by the long-established BTA3D system, additionally showing significantly shorter time to detection.

## MATERIALS AND METHODS

### Study design.

This multicenter blood culture study was a matched-system-design clinical trial comparing four bottle types in the predicate device, BTA3D, with the investigational device, Virtuo. The study design was based on a United States Food and Drug Administration (U.S. FDA) guidance document published in August 1991 (14). The bottle types studied were BacT/Alert SA (aerobic) and BacT/Alert SN (anaerobic) bottles and BacT/Alert FA Plus (aerobic) and BacT/Alert FN Plus (anaerobic) bottles; the latter two bottle types contain adsorbent polymeric beads for neutralization of antimicrobials, while SA and SN bottles contain neither charcoal nor polymeric beads. FA Plus and FN Plus bottles were studied at all three study sites, while SA and SN bottles were studied at one site (Table 1). Patients suspected of having bacteremia or fungemia were studied. The bottle types were studied sequentially by adding a study bottle to each pair of aerobic and anaerobic bottles routinely collected, with each of the bottle types tested over different time periods. Pairs of each bottle type under investigation (i.e., aerobic pairs or anaerobic pairs) were inoculated with a target volume of up to 10 ml of blood each and regarded as study test pairs; no minimum blood volume was required. Additional bottles were also inoculated from the same blood draw as required by each site's routine blood culture protocol; these bottles were not included in the study. Each test pair of aerobic or anaerobic bottles was split between the Virtuo and BTA3D instruments. The order in which the test pairs were inoculated, as well as the order in which they were placed on the instruments, was randomized. Blood culture bottle test pairs were defined as volume compliant with the clinical trial protocol if the volume of blood in both bottles was ≤10 ml/bottle and if the blood volume in the bottle with the smaller volume was within 30% of the blood volume of the bottle with the larger volume. Results from the same bottle type pairs in the two instruments were compared, with results of bottle subcultures classified into significant positives, contaminants, or indeterminate as described below. The target number of volume-compliant, significantly positive culture pairs was set at 200, and it was estimated that approximately 5,500 culture pairs would be required to reach this target. The study was approved by the Institutional Review Board at each study center.

### Blood culture collection and handling.

Blood was obtained using sterile technique by phlebotomists, nurses, physicians, and other health care workers assigned to perform blood cultures for inpatients in wards and intensive care units. No patient exclusion criteria, such as new onset of sepsis or absence of antimicrobial administration, were used. Each bottle was sequentially inoculated with a target of up to 10 ml of blood. Bottles were weighed before blood inoculation, with this weight recorded on the bottle label, and after inoculation. The blood volume in each bottle was calculated from the weight difference in grams divided by 1.04 to adjust for the weight of blood (with each milliliter of blood weighing 1.04 g). The bottles were then loaded into the BTA3D or Virtuo instrument according to the randomization system. Bottles remained in the BTA3D and Virtuo instruments for a maximum of 5 days, unless a positive signal was detected. Bottles giving a positive signal were processed according to each laboratory's standard operating procedure, and time to positivity was recorded. All microorganisms recovered from any bottle from either instrument were saved in a −70°C freezer.

When growth was detected and confirmed (from either instrument) from only one bottle of the test pair and the other bottle in that test pair was not signaled as a suspected positive by the end of the incubation period, the negative bottle in the pair was terminally subcultured to a blood agar plate, incubated in CO_2_, and on an anaerobic blood agar plate, incubated anaerobically. All plates were incubated for a minimum of 72 h at 35 to 37°C to determine if the terminally subcultured bottle represented a false negative. Bottles that gave a positive signal during the 5-day test period with no organisms seen in the Gram stain were subcultured as described above and then reloaded into their respective instruments within 2 h and incubated until growth occurred on subculture, the bottles gave another positive signal, or the original 5-day incubation period expired. In addition, approximately 67% of randomly selected negative-bottle test pairs from both systems were terminally subcultured after 5 days as described above.

### Interpretation of results.

The final culture bottle status interpretation of test pairs tested in both the BTA3D System and Virtuo System was defined as follows:

### Negative.

Bottles that gave a negative signal by either instrument during the 5-day incubation period and that, if subcultured, did not grow an organism on subculture were classified as representing a negative result.

### Positive.

Bottles that gave a positive signal by either instrument when viable organisms were present in the bottle were classified as representing a positive result.

### False positive.

Bottles that gave a positive signal by either instrument when no viable organisms were present in the bottle were classified as representing a false-positive result.

### False negative.

Bottles that gave a negative signal by either instrument when viable organisms were present on subculture of the bottle were classified as representing a false-negative result.

### Significance of positive blood culture isolates.

The significance of positive blood culture isolate results was determined by an independent infectious disease clinician or clinical microbiologist at each trial site by performing a patient chart review in conjunction with the definitions shown below to determine clinical significance. These definitions are based on FDA guidance (14) and recommendations in the literature (12, 15–19).

### Significant.

Results from one or both bottles of the study set that were positive with one or more typical pathogens and that were consistent with the patient's clinical findings were classified as representing significant results. Multiple blood culture sets positive for unusual or typically environmental species were also considered significant.

### Contaminant.

The presence of one of the organisms listed below in a single blood culture bottle or blood culture set from the same draw and in which no other cultures were positive for the same organism from blood collected within 96 h (i.e., 48 h before or after) of the index positive blood culture was considered to represent the presence of a contaminant. Single isolations of the following organisms were generally considered contaminants and typically included about 90% of contaminants as follows: coagulase-negative staphylococci (CoNS), with the exception of S. lugdunensis; Propionibacterium spp.; Bacillus spp. other than B. anthracis; Corynebacterium spp. (diphtheroids); Aerococcus-like organisms; Micrococcus spp.; viridans group streptococci other than S. pneumoniae; and Neisseria spp. other than N. gonorrhoeae or N. meningitidis.

### Indeterminate.

A result with insufficient information available for classification, e.g., the presence of one positive bottle with a likely contaminant where only one set of bottles had been obtained from the patient and clinical relevance could not be determined from the patient's clinical course, was considered to represent an indeterminate result.

### Instrument quality control (QC).

Internal QC was performed by both instruments. BTA3D checks the calibration of each empty bottle cell by measuring the reflectance of an internal cell flag. Measurements outside preset limits require the user to insert four reference standards to recalibrate the cell. Virtuo checks calibration of each cell by comparing optical readings to a calibrated reference standard that is automatically inserted into an empty bottle cell. Measurements outside set limits prompt automatic insertion of four reference standards to recalibrate the cell. For testing of aerobic bottle sets, daily QC was performed in the Virtuo system by testing one positive-control bottle using Pseudomonas aeruginosa alternating with Escherichia coli seeded into a test bottle plus an uninoculated negative-control bottle. In testing anaerobic bottle sets, daily QC was performed in the Virtuo system using Clostridium perfringens alternating with E. coli as well as an uninoculated negative-control bottle. All seeded bottles were expected to give positive signals and uninoculated bottles negative signals. Seed organisms were provided as Bioballs (bioMérieux) containing 30 (± 10%) CFU each; Bioballs were reconstituted in 1 ml of sterile rehydration fluid, which was inoculated into each bottle.

### Data management.

A bioMérieux Web-based data collection and management report system, the BacT/Alert integrated system (BTAIS), was used for manual data entry and for data import from the Virtuo and BTA3D instruments. All patient information was encoded by the software and password protected to prevent unauthorized access. Each site was able to view its own unencoded data, while bioMérieux personnel were able to view encrypted data only.

### Data analysis and statistical methods.

The primary objective of this clinical trial was to demonstrate that, using the same FDA-cleared (or CE-marked) culture bottles, the Virtuo system provides performance substantially equivalent to the performance obtained using the BTA3D system. Data analysis was performed by bioMérieux Biomathematics personnel using SAS statistical software (Version 9.1 or higher). The performances of the test bottle pairs tested in Virtuo and BTA3D systems were compared in two ways. The first compared the system results to the clinical determination results. The second compared the system results to the corresponding terminal subculture results.

## Supplementary Material

Supplemental material
