# Supplementary material for: Multicenter Clinical Evaluation of BacT/Alert Virtuo Blood Culture System
Source: J Clin Microbiol. 2017 Jul 25;55(8):2413–21. doi: 10.1128/JCM.00307-17 (PMC5527419; doi:10.1128/JCM.00307-17)
Supplement: Supplemental material [file supp_55_8_2413__index.html]

Supplemental material 

# Multicenter Clinical Evaluation of BacT/Alert Virtuo Blood Culture System

## Supplemental material

- Supplemental file 1 -

  Table S1 (Numbers of clinically significant isolates by species and recognition by instrument and by volume compliance group)

  PDF, 496K
