# Supplementary material for: Multicenter Clinical Evaluation of BacT/Alert Virtuo Blood Culture System
Source: J Clin Microbiol. 2017 Jul 25;55(8):2413–21. doi: 10.1128/JCM.00307-17 (PMC5527419; doi:10.1128/JCM.00307-17)
Supplement: Supplemental material [file JCM.00307-17_zjm999095591s1.pdf]

Supplemental Table S1. Numbers of clinically significant isolates by species and recognition by instrument, VIRTUO and/or BTA3D, and by volume compliance group.

		Volume compliance status of bottle pairs									All bottle pairs		
		**Compliant**			**Non-compliant, ≤10mL**			**Non-compliant, >10mL**					
**Organism group**	**Organism species**	**Significant positives**	**Detected by both instruments**	**Detected by VIRTUO only ; BTA3D only**	**Significant positives**	**Detected by both instruments**	**Detected by VIRTUO only ; BTA3D only**	**Significant positives**	**Detected by both instruments**	**Detected by VIRTUO only ; BTA3D only**	**Significant positives**	**Detected by both instruments**	**Detected by VIRTUO only ; BTA3D only**
Staphylococci	*Staphylococcus aureus*	45	31	8 ; 6	15	11	2 ; 2	12	9	1 ; 2	72	51	11 ; 10
	Staphylococcus epidermidis	23	12	4 ; 7	10	6	1 ; 3	8	4	3 ; 1	41	22	8 ; 11
	Staphylococcus spp., other	8	5	1 ; 2	2	1	1 ; 0	1	1	0 ; 0	11	7	2 ; 2
Streptococci	*Streptococcus agalactiae*	2	2	0 ; 0	2	2	0 ; 0	3	2	0 ; 1	7	6	0 ; 1
	Streptococcus pyogenes	1	0	1 ; 0	1	1	0 ; 0	3	2	0 ; 1	5	3	1 ; 1
	Streptococcus anginosus/constellatus	4	3	0 ; 1	3	2	1 ; 0	2	2	0 ; 0	9	7	1 ; 1
	Streptococcus gallolyticus	2	2	0 ; 0	2	2	0 ; 0	1	1	0 ; 0	5	5	0 ; 0
	Streptococcus, beta-hemolytic, group C/G	-[a]	-	-	2	0	1 ; 1	-	-	-	2	0	1 ; 1
	Streptococcus, viridans group, other	1	0	0 ; 1	-	-	-	-	-	-	1	0	0 ; 1
	Streptococcus pneumoniae	3	3	0 ; 0	1	0	1 ; 0	-	-	-	4	3	1 ; 0
Enterococci	*Enterococcus durans*	1	1	0 ; 0	-	-	-	-	-	-	1	1	0 ; 0
	Enterococcus faecalis	21	19	1 ; 1	3	3	0 ; 0	7	4	3 ; 0	31	26	4 ; 1
	Enterococcus faecium	6	4	0 ; 2	1	0	1 ; 0	2	0	1 ; 1	9	4	2 ; 3
	Enterococcus gallinarum	1	0	1 ; 0	-	-	-	-	-	-	1	0	1 ; 0
Gram positive bacilli	*Corynebacterium spp.*	1	1	0 ; 0	-	-	-	-	-	-	1	1	0 ; 0
	Janibacter spp.	-	-	-	-	-	-	1	0	1 ; 0	1	0	1 ; 0
	Lactobacillus spp.	1	0	1 ; 0	-	-	-	-	-	-	1	0	1 ; 0
Enterobacteriaceae	Enterobacter aerogenes	2	2	0 ; 0	-	-	-	1	0	1 ; 0	3	2	1 ; 0
	Enterobacter asburiae	2	2	0 ; 0	-	-	-	-	-	-	2	2	0 ; 0
	Enterobacter cloacae	5	3	2 ; 0	3	2	0 ; 1	2	1	1 ; 0	10	6	3 ; 1
	Escherichia coli	33	22	4 ; 7	12	8	3 ; 1	7	5	0 ; 2	52	35	7 ; 10
	Hafnia alvei	-	-	-	-	-	-	1	1	0 ; 0	1	1	0 ; 0
	Klebsiella oxytoca	2	2	0 ; 0	-	-	-	-	-	-	2	2	0 ; 0
	Klebsiella pneumoniae	16	15	1 ; 0	5	0	3 ; 2	3	3	0 ; 0	24	18	4 ; 2
	Proteus spp.	4	2	2 ; 0	1	0	0 ; 1	-	-	-	5	2	2 ; 1
	Salmonella spp.	2	2	0 ; 0	-	-	-	-	-	-	2	2	0 ; 0
Other Gram negative bacilli	Haemophilus influenzae	1	0	0 ; 1	-	-	-	-	-	-	1	0	0 ; 1
	Achromobacter xylosoxidans	-	-	-	-	-	-	1	1	0 ; 0	1	1	0 ; 0
	Acinetobacter baumannii	5	4	1 ; 0	-	-	-	1	0	0 ; 1	6	4	1 ; 1
	Acinetobacter radioresistens	1	0	1 ; 0	-	-	-	-	-	-	1	0	1 ; 0
	Moraxella nonliquefaciens	2	2	0 ; 0	-	-	-	-	-	-	2	2	0 ; 0
	Pseudomonas aeruginosa	5	2	1 ; 2	-	-	-	3	1	1 ; 1	8	3	2 ; 3
	Sphingomonas paucimobilis	1	0	1 ; 0	-	-	-	-	-	-	1	0	1 ; 0
Mycobacteria	Mycobacterium fortuitum	1	1	0 ; 0	-	-	-	-	-	-	1	1	0 ; 0
Gram negative anaerobes	Bacteroides fragilis	2	1	0 ; 1	-	-	-	1	1	0 ; 0	3	2	0 ; 1
	Fusobacterium nucleatum	1	0	0 ; 1	-	-	-	-	-	-	1	0	0 ; 1
	Leptotrichia buccalis	1	0	1 ; 0	-	-	-	-	-	-	1	0	1 ; 0
Gram positive anaerobes	Actinomyces spp.	1	0	1 ; 0	-	-	-	-	-	-	1	0	1 ; 0
	Clostridium spp.	1	0	0 ; 1	-	-	-	-	-	-	1	0	0 ; 1
	Propionibacterium acnes	1	0	0 ; 1	-	-	-	-	-	-	1	0	0 ; 1
Yeast	Candida albicans	7	5	2 ; 0	2	1	0 ; 1	17	10	5 ; 2	26	16	7 ; 3
	Candida glabrata	-	-	-	-	-	-	2	0	2 ; 0	2	0	2 ; 0
	Candida parapsilosis	1	1	0 ; 0	-	-	-	-	-	-	1	1	0 ; 0
Total		217	149	34 ; 34	65	39	14 ; 12	79	48	19 ; 12	361	236	67 ; 58

[a]No isolates of this species from this volume compliance group
